# Correction: The effect of risk framing on support for restrictive government policy regarding the COVID-19 outbreak

**DOI:** 10.1371/journal.pone.0271142

**Published:** 2022-07-05

**Authors:** Kirill Chmel, Aigul Klimova, Nikita Savin

The affiliation for the third author is incorrect. Nikita Savin is not affiliated with #3 but with #2: Department of Integrated Communications, Faculty of Communications, Media, and Design, HSE University, Moscow, Russian Federation.
