# Supplementary material for: The effect of risk framing on support for restrictive government policy regarding the COVID-19 outbreak
Source: PLoS One. 2021 Oct 1;16(10):e0258132. doi: 10.1371/journal.pone.0258132 (PMC8486149; doi:10.1371/journal.pone.0258132)
Supplement: S1 File — (DOCX) [file pone.0258132.s001.docx]

# **S1 File. Eligibility and baseline sample characteristics**

## S1.1. Experiment 1: eligibility and sample characteristics

As it is mentioned in the main text, only current undergraduate or graduate students of the *[anonymized for review]* were eligible to take part in the experiment, except from students of particular departments of Faculty of Social Sciences^[[1]](#footnote-1)^. Since we did not intend to estimate any kind of heterogeneous treatment effect beyond the variation which we could obtain from a student sample, we decided to use a convenience sample, which does not appear to consistently generate false negatives, false positives, or inaccurate effect sizes (Mullinix et al., 2015).

We asked the participants who entered the online platform “At the moment, are you a student of a bachelor, master or graduate level at *[anonymized for review]*?”. As the result, 118 prospective participants were screened out prior to the randomization, since they did not fit the eligibility criteria (7.24% of all who followed the link, N*_All_* =1,629). No aspects of recruitment changed after recruitment began. The calculation of response rate, including the completion rate and the break-off rate, is shown in the main text. Baseline sample characteristics are given in Table 1.

**Table 1.** Baseline characteristics in the final sample (N=762).

| **Variables** | **Categories** | | **N (%) / M (SD)** |
| --- | --- | --- | --- |
| Age | --- | | 21.13 (1.74) |
| Sex | Male | | 167 (21.92) |
|  | Female | | 562 (73.75) |
|  | *NA* | | *33 (4.33)* |
| Degree program | Undergraduate | | 673 (88.32) |
|  | Graduate | | 56 (7.35) |
|  | *NA* | | 33 (*4.33*) |
| Campus | Moscow | | 522 (68.51) |
|  | Saint Petersburg | | 117 (15.35) |
|  | Nizhniy Novgorod | | 44 (5.77) |
|  | Perm | | 46 (6.04) |
|  | *NA* | | *33 (4.33)* |
| Faculty | Math, Electronics and Physics | | 4 (0.53) |
|  | Computer Science | | 59 (7.74) |
|  | Economics. Business and Management | | 172 (22.57) |
|  | Humanities and Social Sciences | | 267 (35.04) |
|  | Communications, Media and Design | | 162 (21.26) |
|  | Other | | 65 (8.53) |
|  | | *NA* | 33 (*4.33*) |
| *Note:* Mean and standard deviation are given for the ‘Age’ variable only. | | | |

## S1.2. Experiment 2: eligibility and sample characteristics

As it is mentioned in the main text, the second experiment was conducted using a volunteer online access panel managed by Online Market Intelligence (OMI) in Russia. The panel has ISO 20252 certification. OMI consumer panel has more than 700,000 panelists in Russia. They recruit panelists from different sources and aim to represent regular Internet users in Russia, but the panel is not representative. The age and gender distribution in the panel is similar to the distribution of Internet users in Russia. OMI recruits from popular websites, e-mail providers, search engines, social networks, banner networks, and partners’ databases.

**Table 2.** Baseline characteristics in the final sample (N=1,438).

| **Variables** | **Categories** | **N (%) / M (SD)** |
| --- | --- | --- |
| Age | --- | 45.65 (14.08) |
| Sex | Male | 653 (45.41) |
|  | Female | 785 (54.59) |
| Education | Higher Education | 598 (41.59) |
|  | Without Higher Education | 840 (58.41) |
| Federal District | Central | 387 (26.91) |
|  | Northwestern | 142 (9.87) |
|  | Southern | 161 (11.20) |
|  | North Caucasian | 96 (6.68) |
|  | Volga | 283 (19.68) |
|  | Ural | 125 (8.69) |
|  | Siberian | 177 (12.31) |
|  | Far Eastern | 67 (4.66) |
| Locality | Moscow | 148 (10.29) |
|  | Saint-Petersburg | 51 (3.55) |
|  | >1mln inhabitants | 151 (10.50) |
|  | 500k – 1mln inhabitants | 299 (20.79) |
|  | <500k inhabitants | 789 (54.87) |
| *Note:* Mean and standard deviation are given for the ‘Age’ variable only. | | |

Nationally representative “soft” quotas on gender (male – 46.4%; female – 53.6%), age (18-24 a.g.^[[2]](#footnote-2)^ – 7.6%; 25-34 a.g. – 21.0%; 35-44 a.g. – 19.0%; 45-54 a.g. – 17.1%; 55+ a.g. – 35.3%), federal district (Central – 26.8%; Northwestern – 9.4%; Southern – 11.2%; North Caucasian – 6.7%; Volga – 20.1%; Ural – 8.4%; Siberian – 13.2%; Far Eastern – 4.2%), locality (Moscow – 10.3%; Saint-Petersburg – 5.2%; >1mln inhabitants – 10.3%; 500k – 1mln inhabitants – 20.4%; <500k inhabitants – 53.9%), and level of education (with higher education – 41.1%; without – 58.9%) were used. Distribution of population across the Russian Federation was taken from Russian Census, 2010. The targeted sample size was 1,500 respondents, and the actual sample size was 1,570. All panelists older than 18 years were eligible to take part in the survey, so 7 prospective participants were screened out prior to the randomization, since they did not fit the eligibility criteria (accessed for eligibility, N*_All_*=1,945). No aspects of recruitment changed after recruitment began. The calculation of response rate, including the completion rate and the break-off rate, is shown in the main text. Since 8.4% (132) of respondents showed low data quality – quick reading of the vignettes (77; 4.9%) and straightlining in grid questions (76; 4.8%), – they were excluded after the randomization prior to statistical analysis. Baseline sample characteristics are given in Table 2.

1. Students of Political Science and Sociology departments were not invited to participate in the survey under the assumption that they might be overly familiar with the treatment material, which was included in the experiment. [↑](#footnote-ref-1)
2. a.g. – age group. [↑](#footnote-ref-2)
