# Supplementary material for: The effect of risk framing on support for restrictive government policy regarding the COVID-19 outbreak
Source: PLoS One. 2021 Oct 1;16(10):e0258132. doi: 10.1371/journal.pone.0258132 (PMC8486149; doi:10.1371/journal.pone.0258132)
Supplement: S2 File — (DOCX) [file pone.0258132.s002.docx]

# **S2 File. Randomization checks**

## S2.1. Experiment 1: randomization checks

**Table 1.** Randomization checks for the main factor randomization: risk severity (low-risk vs. high-risk).

| **Variable** | **Low-Risk** | | | **High-Risk** | | | **p-value** |
| --- | --- | --- | --- | --- | --- | --- | --- |
|  | **N** | **Mean** | **SD** | **N** | **Mean** | **SD** |  |
| Age | 344 | 21.08 | 1.67 | 383 | 21.17 | 1.80 | 0.522 |
| Female | 346 | 0.75 | 0.44 | 383 | 0.79 | 0.41 | 0.123 |
| Have relatives older than 60 | 346 | 0.18 | 0.39 | 383 | 0.17 | 0.38 | 0.725 |
| Probability of COVID-19 infection | 356 | 29.37 | 25.47 | 391 | 33.34 | 26.68 | **0.038** |
| Scale of COVID-19 in Russia | 364 | 2.98 | 0.86 | 398 | 3.01 | 0.86 | 0.668 |
| Frequency of check-ups | 346 | 3.16 | 0.96 | 383 | 3.09 | 0.95 | 0.305 |
| Government capacity to deal with the pandemic | 362 | 3.74 | 0.96 | 396 | 3.81 | 0.94 | 0.309 |
| Watching pro-government news | 354 | 2.08 | 1.57 | 393 | 2.03 | 1.55 | 0.638 |
| *Note:* The p-values of two-tailed t-Tests are reported in the last column. | | | | | | | |

**Table 2.** Randomization checks for the main factor randomization: object at risk (individual losses vs. losses to others).

| **Variable** | **Individual Losses** | | | **Losses to Others** | | | **p-value** |
| --- | --- | --- | --- | --- | --- | --- | --- |
|  | **N** | **Mean** | **SD** | **N** | **Mean** | **SD** |  |
| Age | 358 | 21.08 | 1.66 | 369 | 21.17 | 1.81 | 0.501 |
| Female | 358 | 0.75 | 0.43 | 371 | 0.79 | 0.41 | 0.219 |
| Have relatives older than 60 | 358 | 0.17 | 0.38 | 371 | 0.19 | 0.39 | 0.653 |
| Probability of COVID-19 infection | 364 | 29.79 | 26.04 | 383 | 33.02 | 26.23 | 0.092 |
| Scale of COVID-19 in Russia | 373 | 3.01 | 0.84 | 389 | 2.98 | 0.87 | 0.676 |
| Frequency of check-ups | 358 | 3.14 | 0.98 | 371 | 3.10 | 0.93 | 0.546 |
| Government capacity to deal with the pandemic | 372 | 3.74 | 0.97 | 386 | 3.81 | 0.94 | 0.283 |
| Watching pro-government news | 367 | 2.01 | 1.53 | 380 | 2.09 | 1.59 | 0.492 |
| *Note:* The p-values of two-tailed t-Tests are reported in the last column. | | | | | | | |

**Table 3.** Randomization checks for all four conditions in a completely randomized 2x2 factorial design.

| **Variable** | **Low-Risk X Individual Losses** | | | **High-Risk X Individual Losses** | | | **Low-Risk X**  **Losses to Others** | | | **High-Risk X**  **Losses to Others** | | | **p-value** |
| --- | --- | --- | --- | --- | --- | --- | --- | --- | --- | --- | --- | --- | --- |
|  | **N** | **Mean** | **SD** | **N** | **Mean** | **SD** | **N** | **Mean** | **SD** | **N** | **Mean** | **SD** |  |
| Age | 172 | 21.08 | 1.83 | 186 | 21.09 | 1.50 | 172 | 21.09 | 1.51 | 197 | 21.24 | 2.04 | 0.777 |
| Female | 172 | 0.72 | 0.45 | 186 | 0.78 | 0.42 | 174 | 0.77 | 0.42 | 197 | 0.81 | 0.40 | 0.265 |
| Have relatives older than 60 | 172 | 0.19 | 0.39 | 186 | 0.16 | 0.36 | 174 | 0.18 | 0.38 | 197 | 0.19 | 0.40 | 0.773 |
| Probability of COVID-19 infection | 175 | 28.30 | 25.59 | 189 | 31.17 | 26.44 | 181 | 30.40 | 25.39 | 202 | 35.36 | 26.80 | 0.061 |
| Scale of COVID-19 in Russia | 180 | 3.00 | 0.87 | 193 | 3.02 | 0.83 | 184 | 2.96 | 0.86 | 205 | 3.00 | 0.89 | 0.941 |
| Frequency of check-ups | 172 | 3.16 | 0.96 | 186 | 3.13 | 1.01 | 174 | 3.16 | 0.96 | 197 | 3.05 | 0.91 | 0.620 |
| Government capacity to deal with the pandemic | 180 | 3.73 | 0.99 | 192 | 3.74 | 0.95 | 182 | 3.75 | 0.94 | 204 | 3.87 | 0.93 | 0.437 |
| Watching pro-government news | 175 | 2.08 | 1.59 | 192 | 1.95 | 1.48 | 179 | 2.08 | 1.56 | 201 | 2.10 | 1.61 | 0.781 |
| *Note:* The p-values of F-tests for joint orthogonality are reported in the last column. | | | | | | | | | | | | | |

## S2.2. Experiment 2: randomization checks

**Table 4.** Randomization checks for the main factor randomization: risk severity (low-risk vs. high-risk).

| **Variable** | **Low-Risk** | | | **High-Risk** | | | **p-value** |
| --- | --- | --- | --- | --- | --- | --- | --- |
|  | **N** | **Mean** | **SD** | **N** | **Mean** | **SD** |  |
| Age | 708 | 45.17 | 14.10 | 730 | 46.12 | 14.04 | 0.198 |
| Female | 708 | 0.55 | 0.50 | 730 | 0.55 | 0.50 | 0.957 |
| Higher education | 708 | 0.41 | 0.49 | 730 | 0.42 | 0.49 | 0.492 |
| Take measures to prevent COVID-19 spread | 708 | 0.78 | 1.09 | 730 | 0.77 | 1.05 | 0.785 |
| Afraid of getting sick with COVID-19 | 708 | 4.92 | 1.56 | 730 | 4.85 | 1.58 | 0.436 |
| Scale of COVID-19 in Russia | 708 | 3.16 | 1.29 | 730 | 3.23 | 1.30 | 0.284 |
| Personal health evaluation | 706 | 2.63 | 0.80 | 729 | 2.60 | 0.77 | 0.611 |
| Attitudes to the government first-wave policy | 708 | 15.66 | 5.84 | 730 | 15.78 | 5.77 | 0.679 |
| Watching pro-government news | 699 | 3.64 | 2.09 | 721 | 3.72 | 2.06 | 0.474 |
| Schwartz’s values: Benevolence | 703 | 0.31 | 0.81 | 723 | 0.36 | 0.85 | 0.270 |
| Schwartz’s values: Universalism | 703 | 0.57 | 0.68 | 723 | 0.58 | 0.72 | 0.823 |
| *Note:* The p-values of two-tailed t-Tests are reported in the last column. | | | | | | | |

**Table 5.** Randomization checks for the main factor randomization: object at risk (individual losses vs. losses to others).

| **Variable** | **Individual Losses** | | | **Losses to Others** | | | **p-value** |
| --- | --- | --- | --- | --- | --- | --- | --- |
|  | **N** | **Mean** | **SD** | **N** | **Mean** | **SD** |  |
| Age | 718 | 46.13 | 14.37 | 720 | 45.18 | 13.77 | 0.202 |
| Female | 718 | 0.55 | 0.50 | 720 | 0.54 | 0.50 | 0.912 |
| Higher education | 718 | 0.44 | 0.50 | 720 | 0.39 | 0.49 | **0.049** |
| Take measures to prevent COVID-19 spread | 718 | 0.78 | 1.09 | 720 | 0.77 | 1.05 | 0.969 |
| Afraid of getting sick with COVID-19 | 718 | 4.88 | 1.59 | 720 | 4.88 | 1.55 | 0.963 |
| Scale of COVID-19 in Russia | 718 | 3.18 | 1.31 | 720 | 3.22 | 1.28 | 0.617 |
| Personal health evaluation | 717 | 2.62 | 0.79 | 718 | 2.61 | 0.78 | 0.850 |
| Attitudes to the government first-wave policy | 718 | 15.47 | 5.79 | 720 | 15.97 | 5.81 | 0.104 |
| Watching pro-government news | 710 | 3.64 | 2.08 | 710 | 3.73 | 2.07 | 0.421 |
| Schwartz’s values: Benevolence | 713 | 0.34 | 0.85 | 713 | 0.34 | 0.82 | 0.971 |
| Schwartz’s values: Universalism | 713 | 0.60 | 0.71 | 713 | 0.54 | 0.69 | 0.123 |
| *Note:* The p-values of two-tailed t-Tests are reported in the last column. | | | | | | | |

**Table 6.** Randomization checks for all four conditions in a completely randomized 2x2 factorial design.

| **Variable** | **Low-Risk X Individual Losses** | | | **High-Risk X Individual Losses** | | | **Low-Risk X**  **Losses to Others** | | | **High-Risk X**  **Losses to Others** | | | **p-value** |
| --- | --- | --- | --- | --- | --- | --- | --- | --- | --- | --- | --- | --- | --- |
|  | **N** | **Mean** | **SD** | **N** | **Mean** | **SD** | **N** | **Mean** | **SD** | **N** | **Mean** | **SD** |  |
| Age | 362 | 45.88 | 14.46 | 356 | 46.38 | 14.30 | 346 | 44.42 | 13.70 | 374 | 45.88 | 13.81 | 0.285 |
| Female | 362 | 0.54 | 0.50 | 356 | 0.56 | 0.50 | 346 | 0.56 | 0.50 | 374 | 0.53 | 0.50 | 0.831 |
| Higher education | 362 | 0.44 | 0.50 | 356 | 0.45 | 0.50 | 346 | 0.38 | 0.49 | 374 | 0.40 | 0.49 | 0.209 |
| Take measures to prevent COVID-19 spread | 362 | 0.74 | 1.10 | 356 | 0.81 | 1.07 | 346 | 0.83 | 1.08 | 374 | 0.72 | 1.02 | 0.431 |
| Afraid of getting sick with COVID-19 | 362 | 4.94 | 1.60 | 356 | 4.83 | 1.58 | 346 | 4.89 | 1.53 | 374 | 4.87 | 1.58 | 0.803 |
| Scale of COVID-19 in Russia | 362 | 3.13 | 1.32 | 356 | 3.23 | 1.30 | 346 | 3.19 | 1.26 | 374 | 3.24 | 1.31 | 0.665 |
| Personal health evaluation | 361 | 2.65 | 0.79 | 356 | 2.59 | 0.78 | 345 | 2.61 | 0.81 | 373 | 2.62 | 0.76 | 0.831 |
| Attitudes to the government first-wave policy | 362 | 15.54 | 6.00 | 356 | 15.40 | 5.57 | 346 | 15.77 | 5.68 | 374 | 16.15 | 5.93 | 0.322 |
| Watching pro-government news | 358 | 3.76 | 2.10 | 352 | 3.52 | 2.07 | 341 | 3.52 | 2.09 | 369 | 3.92 | 2.03 | **0.023** |
| Schwartz’s values: Benevolence | 360 | 0.31 | 0.83 | 353 | 0.36 | 0.86 | 343 | 0.31 | 0.79 | 370 | 0.36 | 0.85 | 0.748 |
| Schwartz’s values: Universalism | 360 | 0.61 | 0.68 | 353 | 0.60 | 0.73 | 343 | 0.53 | 0.68 | 370 | 0.56 | 0.70 | 0.451 |
| *Note:* The p-values of F-tests for joint orthogonality are reported in the last column. | | | | | | | | | | | | | |
