# Supplementary material for: The effect of risk framing on support for restrictive government policy regarding the COVID-19 outbreak
Source: PLoS One. 2021 Oct 1;16(10):e0258132. doi: 10.1371/journal.pone.0258132 (PMC8486149; doi:10.1371/journal.pone.0258132)
Supplement: S3 File — (DOCX) [file pone.0258132.s003.docx]

# **S3 File. CONSORT flow diagrams**

## S3.1. Experiment 1 CONSORT

End of Survey (n=729)

♦  Dropped out (n=33)

Since these participants completed 80% of the questionnaire, we include them in the analysis (AAPOR, 2016).

Demographics and Media Consumption

Government Capacity to Fight the Virus

Probability of the Further Spread

Support for Restrictive Government Policy

Manipulation Checks

**Low-Risk X Losses to Others**

Allocated to intervention (n=184)

♦ Received allocated intervention (n=184)

♦ Did not receive allocated intervention (n=0)

**High-Risk X Losses to Others**

Allocated to intervention (n=205)

♦ Received allocated intervention (n=205)

♦ Did not receive allocated intervention (n=0)

**Low-Risk X Individual**

Allocated to intervention (n=180)

♦ Received allocated intervention (n=180)

♦ Did not receive allocated intervention (n=0)

**High-Risk X Individual**

Allocated to intervention (n=193)

♦ Received allocated intervention (n=193)

♦ Did not receive allocated intervention (n=0)

Randomized (n=762)

Values, Trust and Political Attitudes

Risk Literacy

Risk Knowledge

Excluded (n=874)

♦  Not meeting inclusion criteria (n=118)

♦  Dropped out at the first screen (n=590)

♦  Dropped out further but prior to randomization (n=166)

Assessed for eligibility (n=1636)

## S3.2. Experiment 2 CONSORT

Values

♦  Excluded from the final analysis (n=107)

N = 1,438 included in the analysis

♦  Dropped out (n=25)

Since these participants completed 80% of the questionnaire, we include them in the analysis (AAPOR, 2016).

End of Survey (n=1545)

Demographics and Media Consumption

Electronic passport

Support for Restrictive Government Policy

Manipulation Checks

**Low-Risk X Losses to Others**

Allocated to intervention (n=376)

♦ Received allocated intervention (n=376)

♦ Did not receive allocated intervention (n=0)

**High-Risk X Losses to Others**

Allocated to intervention (n=413)

♦ Received allocated intervention (n=413)

♦ Did not receive allocated intervention (n=0)

**Low-Risk X Individual**

Allocated to intervention (n=396)

♦ Received allocated intervention (n=396)

♦ Did not receive allocated intervention (n=0)

**High-Risk X Individual**

Allocated to intervention (n=398)

♦ Received allocated intervention (n=398)

♦ Did not receive allocated intervention (n=0)

Randomized (n=1583)

Sputnik Vaccine

Regional authorities

Risk Knowledge and Trust

Excluded (n=362)

♦  Not meeting inclusion criteria (n=7)

♦  Dropped out at the first screen (n=54)

♦  Dropped out further but prior to randomization (n=120)

♦  Quotas were full (n=181)

Assessed for eligibility (n=1945)
