# Supplementary material for: The effect of risk framing on support for restrictive government policy regarding the COVID-19 outbreak
Source: PLoS One. 2021 Oct 1;16(10):e0258132. doi: 10.1371/journal.pone.0258132 (PMC8486149; doi:10.1371/journal.pone.0258132)
Supplement: S4 File — (DOCX) [file pone.0258132.s004.docx]

# **S4 File. Selection and measurement of pre-treatment covariates**

## S4.1. Experiment 1: pre-treatment covariates

| **Variable Name** | Measurement | Selection Criterion |
| --- | --- | --- |
| Gender | Please specify your gender:1. Male2. Female | Women were found to be more compliant with restrictive government measures (Brouard et al., 2020). |
| Have relatives older than 60 | Do people aged 60 and over live with you in the household or not? *1. Yes, they do*  *2. No, they don’t* | It was found that, for instance, mask use is associated with the household composition; the strongest association was belonging to a household with more than two generations living together (Brankston et al., 2021). In other words, it might reflect the concern for the safety of older individuals in households, who are exceptionally vulnerable. |
| Probability of COVID-19 infection | In your opinion, what is the current probability of contracting coronavirus in Russia? If you find it difficult to give an answer, try to approximately estimate this probability. ___________*%* | Perceived risk and fear can significantly increase engagement in preventive behaviors during the novel coronavirus pandemic (Yıldırım et al., 2021). |
| Scale of COVID-19 in Russia | Do you think the scale of the coronavirus problem in Russia is exaggerated or not? *1. Exaggerated*  *2. Rather exaggerated*  *3. Rather not exaggerated*  *4. Not exaggerated* | Earlier studies showed that with the increase of scepticism and feeling that the danger of pandemic is exaggerated there is a decrease in willingness to comply with restrictive government measures (Prati et al., 2011). |
| Frequency of check-ups | How often did you visit a doctor (for example, a therapist, dentist, ENT, etc.) in the last 12 months? *1. Several times a month*  *2. Once a month*  *3. 2-3 times during the year*  *4. Once a year*  *5. Less than once a year / Almost never* | Vulnerability (e.g., poor health) can significantly increase engagement in preventive behaviors during the novel coronavirus pandemic (Yıldırım et al., 2021). In accordance with the ethical standards of the university, we were only able to ask students indirectly about their personal health. |
| Government capacity to deal with the pandemic | Do you think that in the case of a rapid deterioration in the situation with the coronavirus, will the Russian government be able to protect the population of Russia from its spread or not? *1. Definitely yes*  *2. Rather yes*  *3. Partly yes, partly no*  *4. Probably not*  *5. Definitely not* | There are a lot of studies that show that the level of trust and support of government increases compliance with restrictive government measures in times of public health crisis. For instance, there is evidence that trust in government raises compliance (Murphy et al., 2020). Research in policy implementation shows that public confidence in government institutions and knowledge about policies implemented are critical factors for securing compliance (Kim and Oh, 2015). It was also found that there is an association between increased confidence in government to tackle the pandemic and higher self-reported compliance (Wright et al., 2021). |
| Watching pro-government news | Which of the following sources of information do you use? If you use this source of information, how often? - News on Channel One, Russia-1 or NTV *1. Do not use*  *2. Less than 1-2 times a month*  *3. 1 or 2 times a month*  *4. About once a week*  *5. 2-3 times a week*  *6. Every day / almost every day* | It was found that media use affects compliance with health behaviors (Wu and Shen, 2021). In the Russian context, we control for the exposure to state-provided information about the virus. |

## S4.2. Experiment 2: pre-treatment covariates

| **Variable Name** | Measurement | Selection Criterion |
| --- | --- | --- |
| Age | Please provide the year of your birth. Specify the year in a YYYY format, for example, 1967. ___________ *year* | Age was found to be positively associated with complying with the measures (Brouard et al., 2020), and younger people were proved to be the least compliant (Nivette et al., 2021); however, it was found that the elderly are not always compliant as well (Daoust., 2020). Age was not used in the convenience sample because of the high age homogeneity in this group. |
| Gender | Please specify your gender:1. Male2. Female | Women were found to be more compliant with restrictive government measures (Brouard et al., 2020). |
| Higher Education | What is the highest level of education you have attained?No higher education:1. Primary2. Secondary education (school, lyceum, gymnasium)3. Incomplete secondary plus primary vocational (vocational school, professional technical school, industrial school without a secondary education)4. Specialist-secondary or professional technical (professional technical school, technical college, school)5. Incomplete higher education (at least three years at university)Have higher education:6. Higher education7. Academic degree (PhD, Doctor of Science) | Higher education is associated with greater compliance (Bish and Michie, 2010; Brouard et al., 2020). Education was not used in the convenience sample because of the high education homogeneity in this group. |
| Take measures to prevent COVID-19 spread | In the past seven days, have you had to take any of the following preventive measures? *1. Spent more time at home without going outside*  *2. Restricted visits to public places, public events (shopping centers, cafes, cinemas, theaters, etc.)*  *3. Used a medical mask outside the home*  *4. Wash hands frequently with soap / hand sanitizer*  *5. Limited the number of contacts with friends and family* | We control for the level of existing compliance with the protective measures, which to that moment was obligatory in Russia. |
| Afraid of getting sick with COVID-19 | How afraid are you that you or your loved ones may get coronavirus? *1. Not at all afraid*  *2. Not afraid*  *3. Rather not afraid*  *4. Partly afraid, partly not*  *5. Rather afraid*  *6. Afraid*  *7. Very much afraid* | Perceived risk and fear can significantly increase engagement in preventive behaviors during the novel coronavirus pandemic (Yıldırım et al., 2021). |
| Scale of COVID-19 in Russia | Do you think the scale of the coronavirus problem in Russia is exaggerated or not? *1. Exaggerated*  *2. Rather exaggerated*  *3. Partly exaggerated, partly not*  *4. Rather not exaggerated*  *5. Not exaggerated* | Earlier studies showed that with the increase of scepticism and feeling that the danger of pandemic is exaggerated there is a decrease in willingness to comply with restrictive government measures (Prati et al., 2011). |
| Personal health evaluation | How do you assess your state of health today? *1. Very good*  *2. Good*  *3. Satisfactory*  *4. Bad*  *5. Very bad* | Vulnerability (e.g., poor health) can significantly increase engagement in preventive behaviors during the novel coronavirus pandemic (Yıldırım et al., 2021). |
| Attitudes to the government first-wave policy | In your opinion, how did the following institutions cope with preventing the spread of coronavirus in Russia? - President  - Government  - Governor / Head of your region  - Healthcare system of the Russian Federation  - Rospotrebnadzor  - World Health Organization (WHO)  *1. Very good*  *2. Good*  *3. Satisfactory*  *4. Bad*  *5. Very bad* | There are a lot of studies that show that the level of trust and support of government increases compliance with restrictive government measures in times of public health crisis. For instance, there is evidence that trust in government raises compliance (Murphy et al., 2020). Research in policy implementation shows that public confidence in government institutions and knowledge about policies implemented are critical factors for securing compliance (Kim and Oh, 2015). It was also found that there is an association between increased confidence in government to tackle the pandemic and higher self-reported compliance (Wright et al., 2021). |
| Watching pro-government news | Which of the following sources of information do you use? If you use this source of information, how often? - News on Channel One, Russia-1 or NTV *1. Do not use*  *2. Less than 1-2 times a month*  *3. 1 or 2 times a month*  *4. About once a week*  *5. 2-3 times a week*  *6. Every day / almost every day* | It was found that media use affects compliance with health behaviors (Wu and Shen, 2021). In the Russian context, we control for the exposure to state-provided information about the virus. |
