# Supplementary material for: The effect of risk framing on support for restrictive government policy regarding the COVID-19 outbreak
Source: PLoS One. 2021 Oct 1;16(10):e0258132. doi: 10.1371/journal.pone.0258132 (PMC8486149; doi:10.1371/journal.pone.0258132)
Supplement: S5 File — (DOCX) [file pone.0258132.s005.docx]

# **S5 File. DV measurement: support for restrictive government policy**

## S5.1. Experiment 1: DV measurement

**Table 1.** The list of survey items used to construct the dependent variable ‘Support for restrictive government policy’.

| **Item** | **N** | **M (SD)** | **R** |
| --- | --- | --- | --- |
| 1. Temporary suspension of air communication between the Russian Federation and other countries | 762 | 4.3 (0.98) | 0.69 |
| 1. Temporary suspension of transport links with China | 762 | 4.4 (0.88) | 0.64 |
| 1. Medical examination of people entering the border of the Russian Federation | 762 | 4.7 (0.65) | 0.55 |
| 1. Restriction of Entry into the Russian Federation of Chinese Citizens and Foreign Citizens Arriving from China | 762 | 4.5 (0.85) | 0.64 |
| 1. Restriction of entry into the Russian Federation of Italian citizens and foreign citizens arriving from Italy | 762 | 4.5 (0.80) | 0.68 |
| 1. Restriction of entry into the Russian Federation of foreign citizens and stateless persons | 762 | 4.3 (0.98) | 0.66 |
| 1. Quarantined for 14 days upon arrival of Russians from countries with an unfavorable epidemiological situation | 762 | 4.7 (0.65) | 0.65 |
| 1. Recommendations to work from home (remote mode) | 762 | 4.6 (0.75) | 0.57 |
| 1. Recommendations to move education to remote learning format | 762 | 4.5 (0.88) | 0.60 |
| 1. Advice for older people to stay at home | 762 | 4.7 (0.61) | 0.54 |
| 1. Ban on holding mass events for more than 50 people | 762 | 4.6 (0.74) | 0.65 |
| 1. Closure of sports centers, fitness centers, pools | 762 | 4.5 (0.83) | 0.63 |
| 1. Complete closure of Russian borders | 762 | 3.7 (1.31) | 0.65 |
| 1. Complete closure of Moscow | 762 | 3.3 (1.38) | 0.59 |
| 1. Temporary interruption of air and rail communications within the country | 762 | 3.4 (1.27) | 0.62 |
| 1. Temporary quarantine in all Russian settlements | 762 | 4.1 (1.11) | 0.66 |
| 1. Temporary imposition of a state of emergency in the country | 762 | 3.9 (1.20) | 0.51 |
| 1. Mandatory testing of all residents of the territory for the detection of coronavirus | 762 | 3.8 (1.24) | 0.37 |
| 1. Mandatory testing of all migrants for the detection of coronavirus | 762 | 4.2 (1.20) | 0.40 |
| 1. Temporary closure of all cafes and restaurants in the country | 762 | 4.1 (1.09) | 0.65 |
| 1. Temporary cancellation of church services in the country | 762 | 4.7 (0.81) | 0.47 |
| 1. Temporary suspension of public transport in the country | 762 | 3.3 (1.30) | 0.57 |
| *Note:* R - item-total correlation corrected for item overlap and scale reliability. |  |  |  |

## S5.2. Experiment 2: DV measurement

**Table 2.** The list of survey items used to construct the dependent variable ‘Support for restrictive government policy’.

| **Item** | **N** | **M (SD)** | **R** |
| --- | --- | --- | --- |
| 1. Introduction of the requirement for mandatory wearing of masks in public places | 1438 | 3.89 (1.28) | 0.76 |
| 1. Introduction of a self-isolation regime, restrictions on leaving home for the majority of the population | 1438 | 3.14 (1.25) | 0.81 |
| 1. Introduction of a self-isolation regime, restrictions on leaving home for people over 65 | 1438 | 3.69 (1.24) | 0.71 |
| 1. Maintaining a social distance of 1.5-2 meters between people in public places | 1438 | 4.03 (1.11) | 0.69 |
| 1. Temporary suspension of air traffic between the Russian Federation and other countries | 1438 | 3.93 (1.17) | 0.52 |
| 1. Transfer of company employees to a remote mode of work (online) | 1438 | 3.69 (1.16) | 0.72 |
| 1. Transfer of schools and universities to a distance learning format (online) | 1438 | 3.23 (1.37) | 0.71 |
| 1. Temporary restriction of movement in the city / town | 1438 | 2.80 (1.31) | 0.77 |
| 1. Temporary closure of all cafes, restaurants and bars | 1438 | 3.47 (1.29) | 0.73 |
| 1. Introduction of fines for non-compliance with the self-isolation regime | 1438 | 2.74 (1.45) | 0.69 |
| *Note:* R - item-total correlation corrected for item overlap and scale reliability. |  |  |  |
