# Supplementary material for: The effect of risk framing on support for restrictive government policy regarding the COVID-19 outbreak
Source: PLoS One. 2021 Oct 1;16(10):e0258132. doi: 10.1371/journal.pone.0258132 (PMC8486149; doi:10.1371/journal.pone.0258132)

# **S6 File. ANOVA post hoc analyses**

## S6.1. Experiment 1: ANOVA post hoc analysis

Figure 1 summarizes the results of multiple comparisons. We found that there is no evidence for the interaction effect of the main factors i.e., there are no specific patterns in data which make a particular group to be any different form the other ones. Nevertheless, there are statistically significant differences in the support for restrictive government policy for a few pairwise comparisons including Low-risk X Individual losses vs. High-risk X Losses to Others and High-risk X Individual losses vs. Low-risk X Losses to others (see Figure 1).

**Figure 1.** Multiple comparisons: (a) Low-risk X Individual losses vs. High-risk X Individual losses, (b) Low-risk X Individual losses vs. Low-risk X Losses to others, (c) Low-risk X Individual losses vs. High-risk X Losses to Others, (d) High-risk X Individual losses vs. Low-risk X Losses to others, (e) High-risk X Individual losses vs. High-risk X Losses to others, (f) Low-risk X Losses to others vs. High-risk X Losses to others.

##

## S2.2. Experiment 2: ANOVA post hoc analysis

Figure 2 summarizes the results of multiple comparisons. We found that there is no evidence for the interaction effect of the main factors i.e., there are no specific patterns in data which make a particular group to be any different form the other ones. Nevertheless, there are statistically significant differences in the willingness to sacrifice rights for the Low-risk X Losses to others vs. High-risk X Losses to others comparison (see Figure 2).

**Figure F2.** Multiple comparisons: (a) Low-risk X Individual losses vs. High-risk X Individual losses, (b) Low-risk X Individual losses vs. Low-risk X Losses to others, (c) Low-risk X Individual losses vs. High-risk X Losses to Others, (d) High-risk X Individual losses vs. Low-risk X Losses to others, (e) High-risk X Individual losses vs. High-risk X Losses to others, (f) Low-risk X Losses to others vs. High-risk X Losses to others.

*
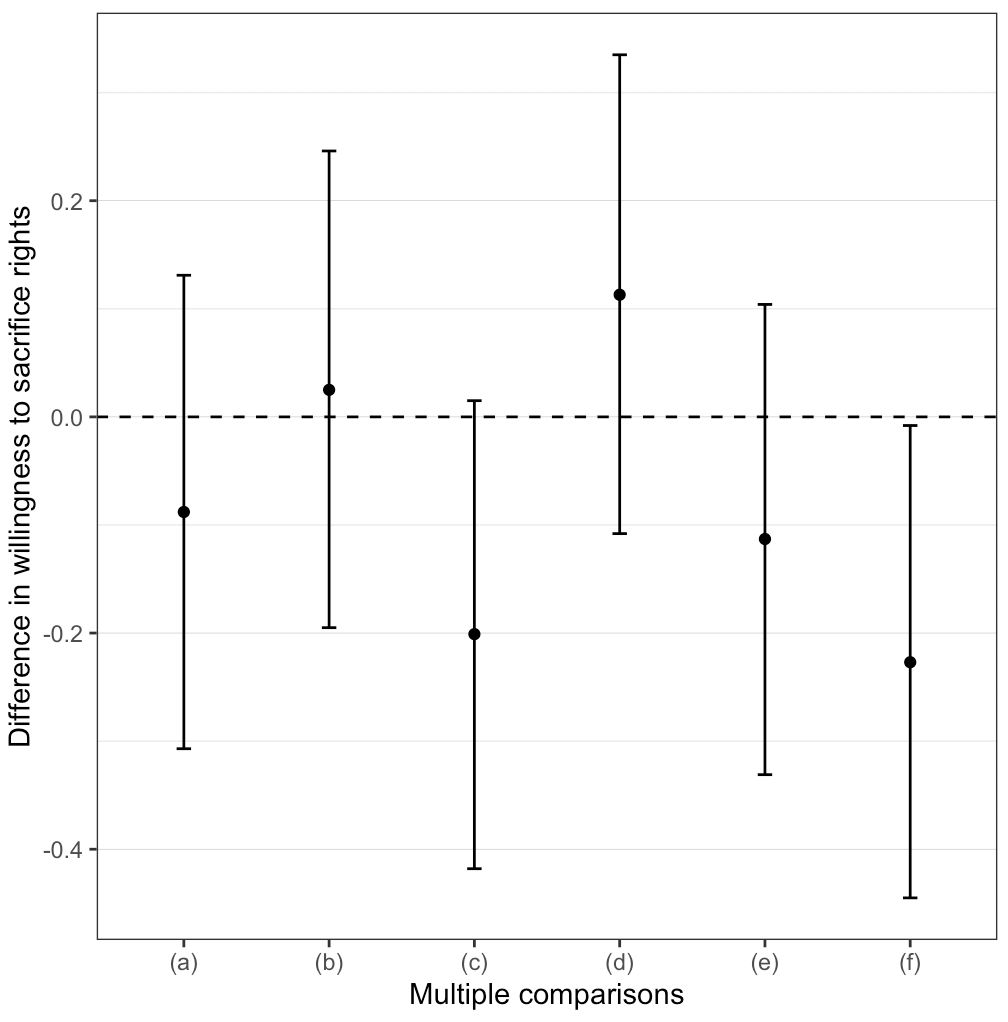
* *
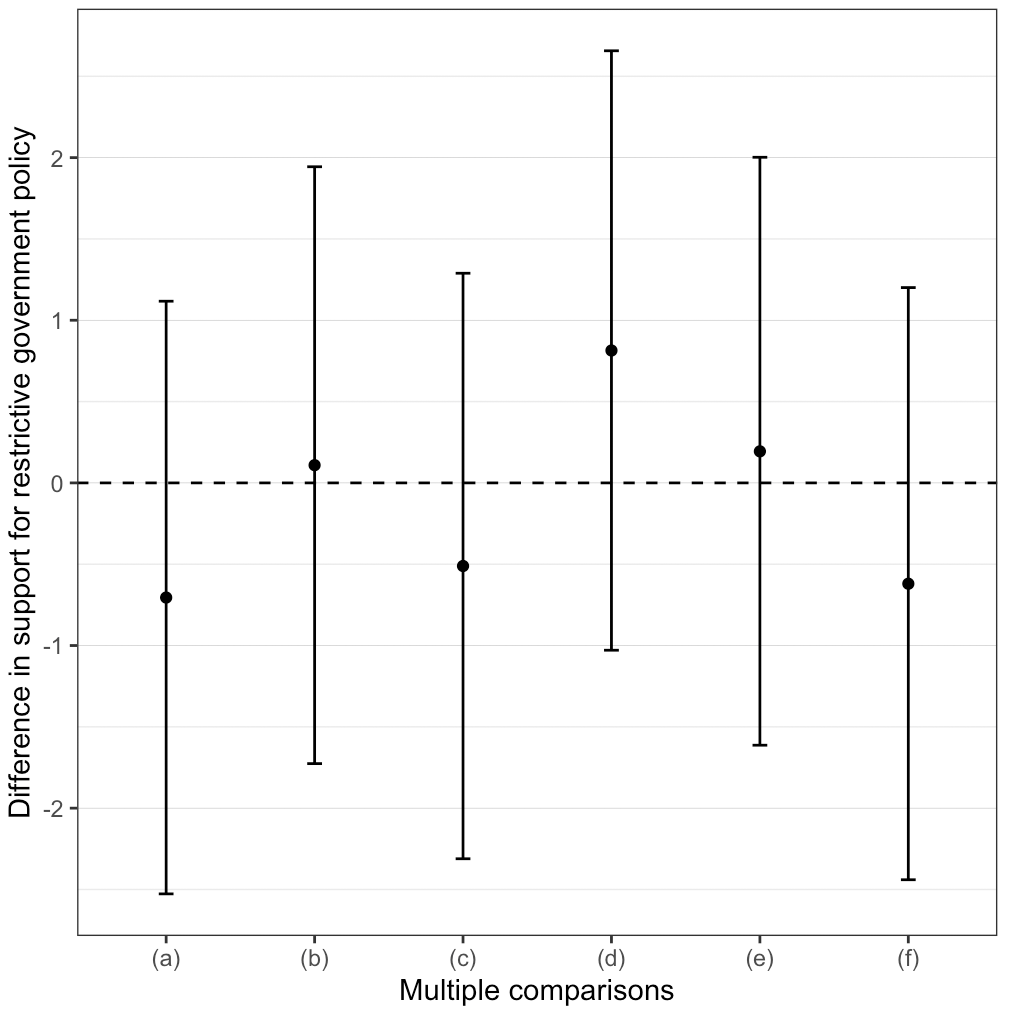
*
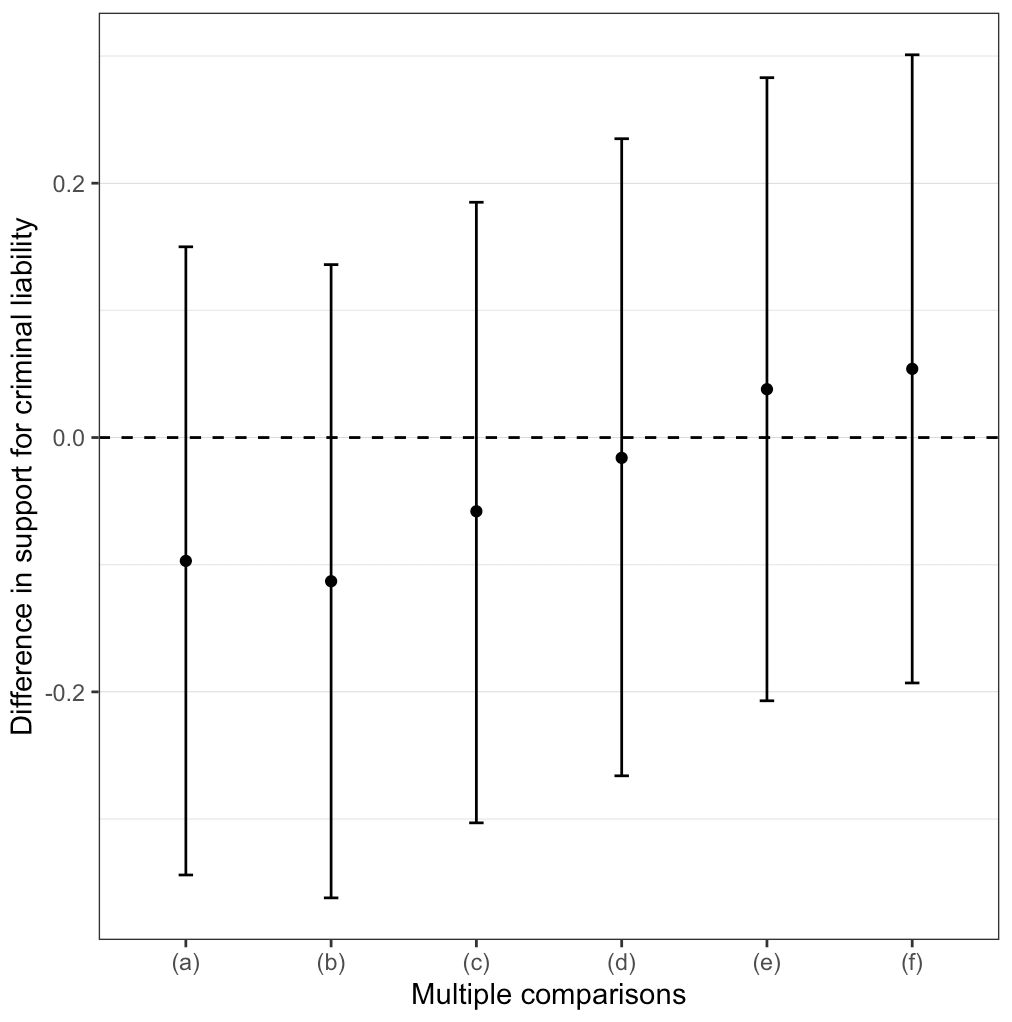

Supplement: S6 File — (DOCX) [file pone.0258132.s006.docx]
