# Supplementary material for: The effect of risk framing on support for restrictive government policy regarding the COVID-19 outbreak
Source: PLoS One. 2021 Oct 1;16(10):e0258132. doi: 10.1371/journal.pone.0258132 (PMC8486149; doi:10.1371/journal.pone.0258132)
Supplement: S7 File — (DOCX) [file pone.0258132.s007.docx]

# **S7 File. Results of OLS models**

## S7.1. Experiment 1: OLS models

**Table 1.** Regression models of experimental treatment (risk severity) and pre-treatment covariates.

|  | | | | | | |
| --- | --- | --- | --- | --- | --- | --- |
|  | Dependent variable: | | | | | |
|  |  | | | | | |
|  | **Willingness to**  **sacrifice rights** | | **Support for restrictive**  **government policy** | | **Support for**  **criminal liability** | |
|  | Model 1 | Model 2 | Model 3 | Model 4 | Model 5 | Model 6 |
|  | | | | | | |
| Intercept | 2.593^***^ | 2.588^***^ | 73.794^***^ | 73.866^***^ | 2.219^***^ | 2.249^***^ |
|  | (0.250) | (0.251) | (3.470) | (3.486) | (0.347) | (0.347) |
| **Factor of Risk Severity: High-Risk** | **0.145^*^** | **0.136^*^** | **3.198^***^** | **3.055^***^** | **0.243^*^** | **0.222^*^** |
|  | **(0.067)** | **(0.067)** | **(0.874)** | **(0.877)** | **(0.096)** | **(0.096)** |
| **Factor of Risk Target: Losses to Others** | **-0.022** | **-0.018** | **-0.096** | **-0.079** | **0.017** | **0.022** |
|  | **(0.066)** | **(0.066)** | **(0.865)** | **(0.869)** | **(0.096)** | **(0.096)** |
| Female | 0.172^*^ | 0.151^†^ | 1.332 | 1.336 | 0.063 | 0.042 |
|  | (0.085) | (0.084) | (1.181) | (1.192) | (0.122) | (0.122) |
| Have relatives older than 60 | -0.046 | -0.049 | -2.235^*^ | -2.354^*^ | 0.020 | 0.010 |
|  | (0.093) | (0.094) | (1.139) | (1.139) | (0.125) | (0.126) |
| Probability of COVID-19 infection | 0.001 | 0.001 | 0.043^**^ | 0.042^**^ | 0.003 | 0.003 |
|  | (0.001) | (0.001) | (0.016) | (0.016) | (0.002) | (0.002) |
| Scale of COVID-19 in Russia | 0.293^***^ | 0.296^***^ | 6.079^***^ | 6.079^***^ | 0.309^***^ | 0.310^***^ |
|  | (0.043) | (0.043) | (0.616) | (0.618) | (0.057) | (0.057) |
| Frequency of check-ups | -0.002 | 0.001 | 0.287 | 0.294 | -0.001 | 0.002 |
|  | (0.035) | (0.035) | (0.491) | (0.493) | (0.051) | (0.051) |
| Government capacity to deal with the pandemic | -0.110^**^ | -0.110^**^ | -1.246^**^ | -1.259^**^ | -0.188^***^ | -0.194^***^ |
|  | (0.039) | (0.039) | (0.477) | (0.479) | (0.052) | (0.052) |
| Watching pro-government news | 0.036^†^ | 0.042^*^ | 0.613^*^ | 0.627^*^ | 0.083^**^ | 0.087^**^ |
|  | (0.020) | (0.020) | (0.262) | (0.264) | (0.031) | (0.031) |
|  | | | | | | |
| N | 715 | 709 | 715 | 709 | 715 | 709 |
| Adjusted R^2^ | 0.103 | 0.104 | 0.213 | 0.212 | 0.080 | 0.080 |
| F Statistic | 10.072^***^ | 10.091^***^ | 22.485^***^ | 22.211^***^ | 7.857^***^ | 7.806^***^ |
|  | | | | | | |
| *Note:* Robust standard errors are given in parentheses as to account for heteroscedasticity. Models 2, 4 and 6 are estimated with the exclusion of influential observations from the sample (according to the leverage and standardized residuals criteria). Variance influence factor (VIF) does not exceed 2 for each of variables in all six models. Significance levels are at ^†^p<0.1; ^*^p<0.05; ^**^p<0.01; ^***^p<0.001. All tests are two-tailed. | | | | | | |

##

## S7.2. Experiment 2: OLS models

**Table 2.** Regression models of experimental treatment (risk severity) and pre-treatment covariates.

|  | | | | | | |
| --- | --- | --- | --- | --- | --- | --- |
|  | *Dependent variable:* | | | | | |
|  |  | | | | | |
|  | **Willingness to**  **sacrifice rights** | | **Support for restrictive**  **government policy** | | **Support for**  **criminal liability** | |
|  | Model 1 | Model 2 | Model 3 | Model 4 | Model 5 | Model 6 |
|  | | | | | | |
| Intercept | 0.472^**^ | 0.513^**^ | 17.541^***^ | 17.422^***^ | 0.589^**^ | 0.512^*^ |
|  | (0.176) | (0.182) | (1.404) | (1.393) | (0.226) | (0.227) |
| **Factor of Risk Severity: High-Risk** | **0.135^**^** | **0.146^**^** | **0.545** | **0.579** | **0.015** | **0.023** |
|  | **(0.051)** | **(0.052)** | **(0.374)** | **(0.378)** | **(0.063)** | **(0.064)** |
| **Factor of Risk Target: Losses to Others** | **0.019** | **0.020** | **-0.463** | **-0.431** | **-0.0001** | **0.0001** |
|  | **(0.051)** | **(0.052)** | **(0.376)** | **(0.380)** | **(0.063)** | **(0.064)** |
| Age | 0.009^***^ | 0.009^***^ | -0.023 | -0.019 | -0.003 | -0.003 |
|  | (0.002) | (0.002) | (0.015) | (0.015) | (0.003) | (0.003) |
| Female | -0.049 | -0.050 | -0.499 | -0.507 | 0.096 | 0.092 |
|  | (0.055) | (0.056) | (0.405) | (0.407) | (0.067) | (0.068) |
| Higher education | 0.001 | -0.005 | -0.874^*^ | -0.788^*^ | -0.092 | -0.087 |
|  | (0.053) | (0.054) | (0.395) | (0.400) | (0.065) | (0.067) |
| Take measures to prevent COVID-19 spread | -0.092^***^ | -0.096^***^ | -1.846^***^ | -1.918^***^ | -0.087^**^ | -0.077^*^ |
|  | (0.026) | (0.026) | (0.204) | (0.208) | (0.031) | (0.031) |
| Afraid of getting sick with COVID-19 | 0.113^***^ | 0.110^***^ | 1.241^***^ | 1.219^***^ | 0.089^**^ | 0.102^***^ |
|  | (0.022) | (0.022) | (0.175) | (0.178) | (0.028) | (0.028) |
| Scale of COVID-19 in Russia | 0.167^***^ | 0.161^***^ | 2.434^***^ | 2.382^***^ | 0.174^***^ | 0.170^***^ |
|  | (0.025) | (0.025) | (0.192) | (0.195) | (0.032) | (0.032) |
| Personal health evaluation | -0.013 | -0.017 | -0.009 | 0.104 | 0.036 | 0.037 |
|  | (0.036) | (0.036) | (0.264) | (0.264) | (0.044) | (0.044) |
| Attitudes to the government first-wave policy | 0.049^***^ | 0.047^***^ | 0.309^***^ | 0.310^***^ | 0.036^***^ | 0.036^***^ |
|  | (0.005) | (0.005) | (0.039) | (0.039) | (0.006) | (0.007) |
| Watching pro-government news | 0.076^***^ | 0.077^***^ | 0.390^***^ | 0.357^***^ | 0.075^***^ | 0.072^***^ |
|  | (0.015) | (0.015) | (0.108) | (0.108) | (0.018) | (0.018) |
|  | | | | | | |
| N | 1,420 | 1,373 | 1,420 | 1,373 | 1,420 | 1,373 |
| Adjusted R^2^ | 0.300 | 0.295 | 0.453 | 0.452 | 0.159 | 0.158 |
| F Statistic | 56.274^***^ | 53.135^***^ | 107.930^***^ | 104.074^***^ | 25.442^***^ | 24.345^***^ |
|  | | | | | | |
| *Note:* Robust standard errors are given in parentheses as to account for heteroscedasticity. Models 2, 4 and 6 are estimated with the exclusion of influential observations from the sample (according to the leverage and standardized residuals criteria). Variance influence factor (VIF) does not exceed 2 for each of variables in all six models. Significance levels are at ^†^p<0.1; ^*^p<0.05; ^**^p<0.01; ^***^p<0.001. All tests are two-tailed. | | | | | | |
