# Supplementary material for: The effect of risk framing on support for restrictive government policy regarding the COVID-19 outbreak
Source: PLoS One. 2021 Oct 1;16(10):e0258132. doi: 10.1371/journal.pone.0258132 (PMC8486149; doi:10.1371/journal.pone.0258132)
Supplement: S8 File — (DOCX) [file pone.0258132.s008.docx]

# **S8 File. Manipulation checks**

## S8.1. Experiment 1: Manipulation checks

We had three manipulation checks. First, to ensure that the independent variable had effectively been manipulated and the participants understood risk framing in the way we wanted them to, we asked them ‘In your opinion, to what extent is the coronavirus dangerous according to the information from the text?’ measuring their perception on a 4-point scale from 1 – ‘Not at all dangerous’ to 4 – ‘Very dangerous’; and a yes-no question ‘In your opinion, is the need to take into account the risks of other citizens to be infected described in the text or not?’. Then, we made a subset of those participants whose answers to these questions match the treatment assigned to them to estimate the average treatment effect on the treated (ATT). As shown in Table 1, the estimates of ATTs are consistent with the estimates of ATEs. The ATT of being exposed to the high-risk framing vs. the low-risk one is 0.275 (*β* = 0.275, *p* < 0.01) on the willingness to sacrifice rights; 4.642 (*β* = 4.642, *p* < 0.001) on the support for restrictive government policy; and 0.373 (*β* = 0.373, *p* < 0.01) on the support of criminal liability for the quarantine violation. We conclude that our results are indeed driven by the risk framing effect, rather than a spurious correlation or an effect of potential confounders.

**Table 1.** Average treatment effects on treated (ATTs).

|  | Dependent variable: | | | | | |
| --- | --- | --- | --- | --- | --- | --- |
|  |  | | | | | |
|  | **Willingness to**  **sacrifice rights** | | **Support for restrictive**  **government policy** | | **Support for**  **criminal liability** | |
|  | Model 1 | Model 2 | Model 3 | Model 4 | Model 5 | Model 6 |
|  | | | | | | |
| High-risk Framing | 0.275^**^ | 0.208^*^ | 4.642^***^ | 3.634^**^ | 0.373^**^ | 0.301^*^ |
|  | (0.088) | (0.085) | (1.251) | (1.130) | (0.125) | (0.126) |
|  | | | | | | |
| Controls |  | ✓ |  | ✓ |  | ✓ |
| N | 426 | 404 | 426 | 404 | 426 | 404 |
| Adjusted R^2^ | 0.020 | 0.166 | 0.031 | 0.254 | 0.018 | 0.104 |
| F Statistic | 9.855^***^ | 9.889^***^ | 14.470^***^ | 16.242^***^ | 8.900^***^ | 6.192^***^ |
|  | | | | | | |
| Note: Standard errors are given in parentheses. Variance influence factor (VIF) does not exceed 2 for each of variables in all six models. Significance levels are at ^*^p<0.1; ^**^p<0.05; ^***^p<0.01. All tests are two-tailed. | | | | | | |

Second, we asked the participants to ‘Rate from 1 - “Very simple to understand” to 4 - “Very hard to understand” how difficult this message seemed to you to understand’ to explore whether there were any differences in the cognitive complexity of treatment materials which were used in the experiment. We did not find statistically significant differences between cognitive complexity means in the high-risk (M = 1.48, SD = 0.6) and the low-risk (M = 1.48, SD = 0.6) groups (t(760) = -0.038, *p* = 0.970), as well as in the ‘individual losses’ (M = 1.44, SD = 0.59) and ‘losses to others’ (M = 1.52, SD = 0.6) groups (t(760) = -1.783, *p* = 0.075). Hence, we assume that cognitive complexity of texts was nearly identical and did not affect the results of the given study.

**Figure 1.** Conditional average treatment effects of high-risk vs. low-risk groups their 95% confidence intervals.

Third, we asked the participants to ‘Rate from 1 - “Definitely not credible” to 4 - “Definitely credible” how credible the information presented in the text is in your opinion’ to see whether there any differences between the perceived credibility of treatment materials. First, the difference in perceived credibility between the ‘individual losses’ (M = 3.01, SD = 0.57) and ‘losses to others’ (M = 3.01, SD = 0.53) groups was proved to be statistically insignificant (t(760) = 0.008, *p* = 0.993). However, we found that the participants who were exposed to the ‘low-risk’ framing (M = 2.91, SD = 0.59) were less convinced of the credibility of the information than those who were in the ‘high-risk group’ (M = 3.1, SD = 0.49), and this difference is statistically significant (t(760) = -4.938, *p* < 0.001). This result is quite anticipated, since previous research has shown that individuals seem to perceive lower risk estimates as less credible and consider such information as ‘less trustworthy’ especially in health communication (Trumbo and McComas, 2003). To account for the confounding effect of information credibility, we estimated conditional average treatment effects (CATE) using the perceived credibility as the moderator of treatment effects. Figure 1 shows that our results are robust to the confounding effect of information credibility. First, we found that the CATEs of risk severity framing increase for those who perceived the information as credible. Second, we also found that for those participants who assumed that the information was ‘definitely credible’ CATEs are particularly strong, as to the effect of high-risk framing vs. low-risk framing for them was positive and statistically significant. At the same time, we also observed that there is a statistically significant but negative treatment effect for those participants who were in the ‘high-risk’ group and considered the information to be ‘definitely not credible’. It does not affect the results of the given study, but it could be a topic for further examination. Since we did not randomize information credibility, we cannot make any conclusions about the mechanism behind that, so we comment on these results regarding the CATEs only.

## S8.2. Experiment 2: Manipulation checks

Similar to the first study, we had three manipulation checks. First, to ensure that the independent variable had effectively been manipulated and the participants understood risk framing in the way we wanted them to, we estimated the average treatment effect on the treated (ATT). As shown in Table 2, the estimates of ATTs are consistent with the estimates of ATEs and even show that there is evidence to support the hypothesis for all three measures. The ATT of being exposed to the high-risk framing vs. the low-risk one is 0.345 (*β* = 0.345, *p* < 0.01) on the willingness to sacrifice rights; 3.425 (*β* = 3.425, *p* < 0.01) on the support for restrictive government policy; and 0.176 (*β* = 0.176, *p* < 0.1) on the support of criminal liability for the quarantine violation. We conclude that our results are indeed driven by the risk framing effect, rather than a spurious correlation or an effect of potential confounders.

Second, we did not find statistically significant differences between cognitive complexity means in the high-risk (*M* = 1.86, *SD* = 0.60) and the low-risk (*M* = 1.84, *SD* = 0.67) groups (*t*(1438) = -0.527, *p* = 0.598), as well as in the ‘individual losses’ (*M* = 1.86, *SD* = 0.65) and ‘losses to others’ (*M* = 1.85, *SD* = 0.67) groups (*t*(1438) = 0.227, *p* = 0.820). Hence, we assume that cognitive complexity of texts was nearly identical and did not affect the results of the given study.

**Table 2.** Average treatment effects on treated (ATTs).

|  | | | | | | |
| --- | --- | --- | --- | --- | --- | --- |
|  | Dependent variable: | | | | | |
|  |  | | | | | |
|  | **Willingness to**  **sacrifice rights** | | **Support for restrictive**  **government policy** | | **Support for**  **criminal liability** | |
|  | Model 1 | Model 2 | Model 3 | Model 4 | Model 5 | Model 6 |
|  | | | | | | |
| High-risk Framing | 0.345^***^ | 0.138^*^ | 3.425^***^ | 1.542^***^ | 0.176^*^ | 0.011 |
|  | (0.090) | (0.079) | (0.726) | (0.552) | (0.102) | (0.097) |
|  | | | | | | |
| Controls |  | ✓ |  | ✓ |  | ✓ |
| N | 653 | 644 | 653 | 644 | 653 | 644 |
| Adjusted R^2^ | 0.020 | 0.302 | 0.032 | 0.482 | 0.003 | 0.164 |
| F Statistic | 14.613^***^ | 28.783^***^ | 22.232^***^ | 60.818^***^ | 2.954^*^ | 13.576^***^ |
|  | | | | | | |
| Note: Standard errors are given in parentheses. Variance influence factor (VIF) does not exceed 2 for each of variables in all six models. Significance levels are at ^*^p<0.1; ^**^p<0.05; ^***^p<0.01. All tests are two-tailed. | | | | | | |

Third, the difference in perceived credibility between the ‘individual losses’ (*M* = 2.84, *SD* = 0.67) and ‘losses to others’ (*M* = 2.85, *SD* = 0.63) groups was proved to be statistically insignificant (*t*(1436) = -0.297, *p* = 0.766). However, we found that the participants who were exposed to the ‘low-risk’ framing (*M* = 2.76, *SD* = 0.65) were less convinced of the credibility of the information than those who were in the ‘high-risk’ group (*M* = 2.92, *SD* = 0.64), and this difference is statistically significant (*t*(1436) = -4.936, *p* < 0.001). Figure 2 shows that our results are robust to the confounding effect of information credibility.

**Figure 2.** Conditional average treatment effects of high-risk vs. low-risk groups their 95% confidence intervals.
