# Supplementary material for: The effect of risk framing on support for restrictive government policy regarding the COVID-19 outbreak
Source: PLoS One. 2021 Oct 1;16(10):e0258132. doi: 10.1371/journal.pone.0258132 (PMC8486149; doi:10.1371/journal.pone.0258132)
Supplement: S9 File — (DOCX) [file pone.0258132.s009.docx]

# **S9 File. References used in supporting information**

1. Bish A, Michie S. Demographic and attitudinal determinants of protective behaviours during a pandemic: A review. British Journal of Health Psychology. 2010;15(4):797-824.
2. Brankston G, Merkley E, Fisman DN, Tuite AR, Poljak Z, Loewen PJ, Greer AL. Socio-demographic disparities in knowledge, practices, and ability to comply with COVID-19 public health measures in Canada. Canadian Journal of Public Health. 2021;112(3):363-375.
3. Brouard S, Vasilopoulos P, Becher M. Sociodemographic and psychological correlates of compliance with the Covid-19 public health measures in France. Canadian Journal of Political Science. 2020;53(2):253-258.
4. Daoust JF. Elderly people and responses to COVID-19 in 27 Countries. PloS One. 2020;15(7).
5. Kim J, Oh SS. Confidence, knowledge, and compliance with emergency evacuation. Journal of Risk Research. 2015;18(1):111-126.
6. Mullinix KJ, Leeper TJ, Druckman JN, Freese J. The generalizability of survey experiments. Journal of Experimental Political Science. 2015;2(2):109-138.
7. Murphy K, Williamson H, Sargeant E, McCarthy M. Why people comply with COVID-19 social distancing restrictions: Self-interest or duty?. Australian and New Zealand Journal of Criminology. 2020;53(4):477-496.
8. Nivette A, Ribeaud D, Murray A, Steinhoff A, Bechtiger L, Hepp U, Shanahan L, Eisner M. Non-compliance with COVID-19-related public health measures among young adults in Switzerland: Insights from a longitudinal cohort study. Social Science and medicine. 2021;268:113370.
9. Prati G, Pietrantoni L, Zani B. Compliance with recommendations for pandemic influenza H1N1 2009: the role of trust and personal beliefs. Health Education Research. 2011;26(5):761-769.
10. Wright L, Steptoe A, Fancourt D. Predictors of self-reported adherence to COVID-19 guidelines. A longitudinal observational study of 51,600 UK adults. The Lancet Regional Health - Europe. 2021;4:100061.
11. Wu Y, Shen F. Exploring the impacts of media use and media trust on health behaviors during the COVID-19 pandemic in China. Journal of Health Psychology. 2021.
12. Yıldırım M, Geçer E, Akgül Ö. The impacts of vulnerability, perceived risk, and fear on preventive behaviours against COVID-19. Psychology, Health and Medicine. 2021;26(1):35-43.
