# Supplementary material for: The effect of risk framing on support for restrictive government policy regarding the COVID-19 outbreak
Source: PLoS One. 2021 Oct 1;16(10):e0258132. doi: 10.1371/journal.pone.0258132 (PMC8486149; doi:10.1371/journal.pone.0258132)
Supplement: Attachment — Submitted filename: Response_to_Reviewers.docx [file pone.0258132.s010.docx]

*27 June 2021*

Dear Editorial Board,

We thank all three Reviewers and the Editor for their generous comments and suggestions on the manuscript. We have edited the manuscript to address their concerns. In this rebuttal letter we describe the changes made in response to the reviewers’ comments below. We also attach the revised manuscript and the revised version of supplementary materials with highlighted changes.

**Response to the Reviewer #1**

*This study tested the effect of severity of risk description (low vs. high) and object at risk (self vs. others) on people’s attitude toward government restrictive policies. With two experiments at two different stages during the covid pandemic, the study found the severity of risk was consistently significant. On the other hand, the effect of object at risk was moderated by prosocial values. This study has multiple positive features. Its topic has an important practical implication. The sample size was relatively large. The research questions were tested twice in different stages. The main text was concise but still informative (I like the comprehensive information in the appendix). Overall, I believe the conclusion of this study was supported by its methodology and analyses. I have a few questions and I hope the authors can clarify.*

*1) First, this study tested issue framing and the key manipulation was severity of the risk. Based on my reading of your materials, it seems higher risk condition was associated with more significant losses. As a result, you did find that higher risk led to more positive ratings on the government restrictive policies. Hence, it appears more losses might cause people to take actions to mitigate the spread of the virus (i.e., function of loss aversion). Interestingly, some studies with gain-loss framing found mixed results on the effect of loss aversion (for your reference see a recent study* [*https://psyarxiv.com/4wc5d/*](https://psyarxiv.com/4wc5d/) *This study also tested risk attitude and political ideology as relevant to your study). While your study did not directly test gain-loss framing, can you still discuss some implications on loss aversion and gain-loss framing based on your findings?*

We thank the reviewer for this comment and the proposed reference. Indeed, recent studies address the effects of different framings on following the protective measures during the pandemic. Steffen & Cheng (2021), for instance, work in the paradigm of gain-loss framing. However, there are studies that also tested the framing effect on behaviors and attitudes concerning the COVID-19 pandemic, but these studies did not specifically employ the gain-loss framing (Jordan et al., 2020; Palm et al., 2021). Thanks to the reviewer's comment, we have drawn attention to this and added a short summary of such studies to the Introduction section *(see p. 3)*. This should allow a reader to better contextualize the effect in the literature on framing effects during the COVID-19 pandemic.

We did not explicitly discuss the implications of our analysis for the literature on gain-loss framing in the Discussion section; and we want to clarify our opinion on that. Prospect theory with the gain-loss framing in risk research usually suggests that each decision (e.g., to wear a mask or adopt social distance) has both sides: it has some probability of losses and some probability of gains. As a result, researchers can produce experiments with any decision under study either focusing on possible losses or possible gains when some decision is undertaken. Prospect theory is often applied to the program evaluation (e.g., some programs of saving people’s lives) when the gains vs losses are emphasized or some individual decision with focusing on gains and losses in different treatments.

Our study is based more on the idea of the psychometric paradigm and media frame theories, which makes it different from the framework described above. In our experimental study we did not emphasize any gains and did not introduce any decision. We did not introduce the possible gains of wearing masks, washing hands, adopting social distance, or self-isolation. We framed the COVID-19 pandemic overall (not decision or a policy) as a higher or a lower risk based on the official information and trusted media. To emphasize this, we also slightly changed the structure of the literature review so as not to confuse our experiment with the gain-loss research, and to put more emphasis on the psychometric paradigm *(see p. 4; p. 7)*.

*2) Second, you adopted a typical 2*2 design and I was wondering why you used t-test repeatedly instead of running an anova with post hoc t-tests? Repeat t-test might inflate type I error.*

The reason why we initially excluded the part of the analysis with post hoc tests - they were reported in earlier versions of the manuscript - was our concern about the Type II Error. Multiple comparisons procedure is well known to increase the number of instances in which the null hypothesis is not rejected when in fact it should have been (Gelman, Hill, and Yajima 2012). Hence, we used simple t-tests to show the main effects and then estimated OLS regressions to test for the main effects and the interaction. We do appreciate that the Reviewer noticed that, so to convince a reader we provided information about the results of post hoc analysis in S6 File.

*Relatedly (I was confused here so hope you can clarify), for your anova test, what was the independent variable? For example, you stated “As expected, we found statistically significant differences in the support for restrictive government policy (F(3, 762) = 4.68, p < 0.01, see Table 2, Fig 1) and the support for criminal liability for the quarantine violation (F(3, 762) = 2.72, p < 0.05).” In other words, for these F-tests, which compared to which?*

To determine whether any of the differences between the group means are statistically significant, we first ran one-way ANOVA models in which we used treatment assignment as the independent variable. To clarify that, we rephrased the description of ANOVA models in the following way: “As expected, we found statistically significant differences in the support for restrictive government policy (F(3, 762) = 4.68, *p* < 0.01, see Table 3, Fig 1) and support for criminal liability for the quarantine violation (F(3, 762) = 2.72, *p <* 0.05) between four treatment conditions”. We know that another strategy to analyze 2x2 experiments is to use two-way ANOVA, but we did not use this strategy since we estimated OLS regressions. OLS regressions and two-way ANOVA models are supposed to produce the same estimates of experimental factors effects.

*In the main text you stated there was no interaction. However, looking at Figures 1b, 1c, 2a, and 2c, it seems there are interactions between risk severity and object at risk. Did you test these interactions with either anova or OLS?*

We tested interactions between main factors, and to make the analysis complete, we included this information in the main body of the manuscript as suggested. According to the results of OLS models, interaction effects are not statistically significant neither in Experiment 1 nor in Experiment 2. As for Experiment 1, models 2, 4, and 6 in Table 4 demonstrate that there are no statistically significant interaction effects between risk severity factor and object at risk, neither on willingness to sacrifice rights (β = -0.000, *p* = 0.999), nor on the support for restrictive government policy (β = 1.074, *p* = 0.569), nor on the support for criminal liability for the quarantine violation (β = 0.097, *p* = 0.612). As for Experiment 2, models 2, 4, and 6 in Table 8 demonstrate that there are no statistically significant interaction effects between risk severity factor and object at risk, neither on willingness to sacrifice rights (β = 0.138, *p* = 0.250), nor on the support for restrictive government policy (β = -0.085, *p* = 0.932), nor on the support for criminal liability for the quarantine violation (β = -0.151, *p* = 0.268).

*3) I like your variable of watching pro-government news. Since two of your dependent variables dealt with government policies, was there an interaction between risk severity and watching pro-government news?*

We thank the Reviewer for this interesting question. Though this was not our research question, we checked if there was an interaction effect between risk severity and watching pro-government news as suggested by the Reviewer. We found no statistically significant interaction effects in all three dependent measures while predicting them based on the main factors and variable ‘watching pro-government news’ in OLS regression models:

- *β* = - 0.049, p = 0.261 while predicting willingness to sacrifice rights;
- *β* = - 0.350, p = 0.564 while predicting support for restrictive government measures;
- *β* = - 0.109, p = 0.078 while predicting support for the introduction of criminal liability for violation of the quarantine.

**Response to the Reviewer #2**

*Thank you for the opportunity to review this interesting paper about the effect of risk framing on the support for governmental COVID-19 measures in Russia. This study provides some useful tests for the influence of a high risk versus low risk frame as well as the frame of self-protection compared to the responsibility to protect others on the acceptance of coronavirus protection policies. With help of two different survey experiments, the authors show that by framing the coronavirus as risky and dangerous, the willingness to sacrifice some personal rights for the greater good can be increased. In the course of this, they also show the importance of how credible the information is perceived to be. They do not find support for their hypothesis that framing others to be at risk increases the acceptance of restrictive policy measures. However, they mention evidence that people with prosocial attitudes react to the solidarity treatment, and claim to be more willing to sacrifice own rights for the protection of others.*

*Overall, this survey experiment in Russia provides some interesting and new insights about the acceptance of COVID-19 measures and to what extent the coronavirus support is influenced by different kinds of risk framing. In general, procedure and results are nicely described, there is a great quantity of different analyses and checks, and the study is written very comprehensible. However, there are some aspects that should respectively could be improved - in particular concerning the provision of analyses results and the discussion of the procedure and the results. Since most of the following comments only entail minor revisions, their implementation should be possible without major difficulties.*

*First, I would like to state one major issue concerning the test of hypothesis 2a that needs to be addressed.*

*a) Whereas there is plenty of information (such as descriptive tables, t-tests, and randomization and manipulation checks) in the whole paper and in the supporting information, there is a lack of important information concerning the tests of hypothesis 2a. First of all, for better orientation in the paper, it would be helpful, if it is directly mentioned that hypothesis 2a is studied and discussed now (page 17 and/or in the discussion). More important than this is that the analyses and results of H2a, which lead to the conclusions, need to be sufficiently presented. Although the authors mention the OLS models testing H2a, they do not present enough of the model and results (neither in the text nor in the supporting information). For instance, it is not clear if controls had been included and if so which ones. Also, as a reader I would like to look at the main effects, too, and be able to see the whole picture of main effects and the interaction (which can be enlightening and somewhat surprising as in the case of the credibility interaction in Table 5). Because of this, and since the authors promote the interaction of prosocial persons and the willingness to sacrifice own rights for others as a major finding of the study, I think, they have to present the results of the complete OLS models somewhere (at least in the supporting information).*

We revised the analysis of the hypothesis H2a and included all the sufficient information to interpret the results. First of all, we directly mentioned in the text that we test the hypothesis H2a in this exact place. Second, we added to the main body another table with the results of OLS regression modelling. In this table we demonstrate both main effects and interactions.

*b) Additionally, I missed the presentation of the moderating Schwartz values "Benevolence" and "Universalism" in the supporting information at the randomization checks (chapter B2). However, instead of the moderating variables there is another Schwartz value presented ("Security"), which is not mentioned anywhere else. It would be helpful to be more consistent and more precise about which Schwartz values had been used for which analyses, and why.*

Thank you for this suggestion. We have now included Schwartz’s values ‘benevolence’ and ‘universalism’ in the randomization checks and excluded ‘security’ value. We had also included a description of the Schwartz theory of basic human values, his approach to measurement of values, and the rationale behind the selection of values as moderators in this study on *pp. 7-8*.

*c) In experiment 1, I do not fully understand why hypothesis 2a cannot be tested due to too little variation in the moderating variables. What does this mean here and where can this information be found? For instance, the Schwartz values are not presented in the descriptive table here. Does this mean that these variables had not been conducted in the first experiment and therefore included in the second study to test H2a? Alternatively, had all students (more or less) the same attitudes? If there are further results of this topic, which had not been presented yet, it should be considered to provide them – at least in the supporting information.*

We thank the Reviewer for this comment, and we added more information about the rationale behind not testing hypothesis H2a in the first experiment *(see pp. 10-11)*. Mullinix et al. (2015) in their well-known paper *“The Generalizability of Survey Experiments”* suggest that estimation of CATEs in experimental studies - which is necessary to test our hypothesis H2a - is problematic when there is lack of variance of the moderator variable in convenience samples. Since there is some evidence that the differences among students in prosocial values are not quite large (Hofmann-Towfigh, 2007), we did not test hypothesis H2a in Experiment 1. Hence, we did not include Scwartz's portrait values questionnaire in the questionnaire used in Experiment 1.

*Second, I want to give some comments on minor issues that could help the authors to improve their paper.*

*1) Since this study was carried out in Russia, I suggest adding more detailed information about the situation as well as the measures in this country already in the introduction. In the introduction, countries like Sweden and Austria are mentioned, but not Russia, where the experiments had been carried out. As a reader, I would like to know more COVID-19 in Russia. There is even some information about Russia in the cited Gallup poll. [The cited link to the poll does not work anymore. But, here is one that worked for me:* [*https://www.gallup-international.com/fileadmin/user_upload/surveys/2020/GIA_SnapPoll_2020_COVID_Tables_final.pdf*](https://www.gallup-international.com/fileadmin/user_upload/surveys/2020/GIA_SnapPoll_2020_COVID_Tables_final.pdf)

*Also, the information from the Gallup poll is from the very beginning of the COVID-19 pandemic. I would also like to see some more recent information – ideally from around the time when the second nationwide experiment took place – because it is possible that the attitudes changed from the first to the second wave. Also, the restrictive policy measures changed over, as we can see in the supporting information. However, some more detailed description in the text would be interesting and could help to develop a better understanding of the situation and the results. So, to me, the chapter "Context of the study, COVID-19 in Russia" could be extended or alternatively included in the introduction.*

We do appreciate that there is interest in the context of the study, so we provided more information about the COVID-19 pandemic in Russia. First, we directly mentioned Russia in the Introduction section to compare the statistics in Russia with the situation in the rest of the world *(see p. 3)*. Apart from that, we updated the statistics and mentioned percentages of willingness to sacrifice some of the human rights at the time when both the first and the second experiments were conducted. We also thank the reviewer for noticing that the link has been updated. We took it into consideration and included the new link to the Gallup survey.

Second, the section “The context of the study: COVID-19 in Russia” was extended. We added more information about the restrictive measures which were introduced in times of both the first and the second waves of the COVID-19 in Russia.

*2) The pre-treatment covariates of both experiments are presented in Table 1 and Table 3. However, most seem to be a little lost, since they are not mentioned or explained (such as "Scale of COVID-19 in Russia", "Watching pro-government news" or "Take measures to prevent COVID-19 spread"). I see that they are not of main interest in this study, but some further information would be helpful. Why had just these pre-treatment covariates been included, and considered being important for this study? At least in the supporting information there could be further explanation about the purpose and consequences of these variables and their relevance in terms of the two factors of the survey experiment.*

Despite criticism of the application of the regression approach to randomized control trials (e.g. Humphreys 2009), we ran a number of OLS models to control for pre-treatment covariates and to improve the precision of regression estimates of the experimental treatment effect as the last step of statistical analysis. We have to admit that we did not include the selection criteria of pre-treatment covariates in the main body of the manuscript, since these variables are not the primary interest of this study. We updated the content of supplementary materials and described there in more detail both measurement and selection criteria of all pre-treatment covariates so that readers can understand the rationale for the selection (see S4 File).

*3) In the second experiment, the wording of the vignettes – and therefore the treatment of the study – had been changed. The first question that arises here is: Why? Had there been some issues with the vignettes in the first experiment? At the first glance, manipulation checks (supporting information G1) do not suggest changing the vignettes. So, what were the reasons behind this decision? Second, is it possible that the change of the vignettes affected the results or are there some arguments why this is not likely or why the changing was even necessary? I see that the results of H1 are somewhat ambivalent (there is less support for H1 in the second experiment). There could be various possible reasons for the differences in the results between experiment 1 and 2. They had been carried out at different points of time (first vs. second wave). The population/sample is not the same (student sample vs. nationwide internet users). The treatment had been changed. I think that these decisions need to be justified, and their impact and the arising questions should be discussed.*

We added more explanation of how the vignettes had been changed in Experiment 2 compared to Experiment 1 on *p. 21*. Since this was the second wave of COVID-19 we changed the wording of vignettes. We excluded some basic information which was quite new at the beginning of COVID-19 pandemic (about the pandemic overall, spread of the issue, incubation period), but was common knowledge at the beginning of the second wave. As a result, the number of words has been substantially decreased compared to Experiment 1. Moreover, the statistics have changed since the beginning of COVID-19 pandemic. We described the scale of the issue / recovering, medical treatment, and the advice on how to protect yourself / take care of others. We do agree that the wording of the vignettes can produce differences in effect sizes which can not be completely disentangled from the effect of different time points and survey population. As a result, we have added the limitation of the study on *p.33*.

*4) Discussion, page 20: Unfortunately, I cannot follow how the authors derive that the support for H1 shows that there is more risk averse behavior in the high-risk framing. As far as I understood the study, it was more an effect of willingness to sacrifice rights and support of restrictive measures than actual risk averse behavior (such as staying at home or reducing meetings with others). So, I would appreciate it if the authors would think about their wording here, or could help me understand this conclusion better by some further explanation.*

Thank you for this comment. We have removed risk averse behavior regarding H1. We do agree that the discussion of implications for risk-aversion is misleading and irrelevant for the given study.

*5) There seems to be an issue with the tables in the results part of the second experiment. There are two Table 3 (page 15 and page 16). Table 4 is mentioned in the text on page 18, but the content of this Table 4 is the same as in Table 3 on page 16. So, reading the text, I guess that Table 3 of page 16 is actually Table 4. Table 4 from page 18 can be dropped. Table 5 contains the right information about the credibility interaction.*

Tables were revised. Now, correct tables and proper references to them are used in the manuscript.

*Also, please be aware that basically the same section is accidentally included twice ["In other words, the second experiment demonstrates empirical evidence of the H1 for all three measures of attitudes towards restrictive government policy, but the framing effect of high vs. low risks is observed only for those who perceive information as credible (see main effects and interactions in Table 4/5). On the contrary, in the first study we found that the framing effect was consistent among all respondents, though it was also conditioned by the perceived information credibility."; this section can be found on page 18 before "Table 4" and on page 19 after the end of Table 5.]*

The analysis of the second experiment was revised, so we removed this redundant repetition of the same information.

*6) The citation of Trumbo and McComas (2003) is formally not in harmony with the remaining citations. The resource is mentioned on page 20 and in the supporting information. But this paper is not listed as a resource; hence, it should be added to the references section.*

The reference was added. We also revised the list of references to make sure that all the references are included.

*7) Looking at Figure 2 and reading that there is support for hypothesis 1, it seems that the descriptive statistics of high-risk and low-risk are accidentally interchanged ["… willingness to sacrifice rights between the high-risk (M = 2.88; SD = 1.13) and low-risk (M = 3.04; SD = 1.15) conditions … "] on page 16.*

We thank the Reviewer for such careful reading of the manuscript. Indeed, there was a mistake, and we fixed it in the updated version of the manuscript.

*8) Finally, (though somewhat funny) the typo on page 9 – farming instead of framing – should be corrected.*

We thank the Reviewer for thoroughly reviewing the manuscript and noticing even minor typos and grammar mistakes. The updated version of the manuscript was revised by a native English speaker in two proofreading sessions.

**Response to the Reviewer #3**

*This paper examines the effects of message framing on support for COVID-related restrictions. Overall, while the theoretical contribution is not new, I do believe that the studies provide useful insights into the efficacy of message framing approaches in Russia during the pandemic. My suggestions primarily involve improving the clarity of paper.*

*- Because there are now a number of published and working papers on the effects of COVID-related message framing, it would help to contextualize the current work by more thoroughly summarizing the related literature.*

We thank the reviewer for this comment and admit that this suggestion is in line with the comments made by the Editor and the Reviewer #1. In the revised version of the manuscript we discuss a number of papers on the effect of different COVID-related message framings on promoting self-care behaviors. We added a short summary of these studies to the Introduction section *(see p. 3).* We also put an additional focus on the recent studies which showed the effect of prosocial messages on pandemic response. This literature was added on *pp.6-7* in the sub-section “Who is the main object at risk?” and *p. 30* in the Discussion. We included all papers suggested by the Editor and we found some other papers.

*- In addition, I appreciated the detailed web appendix. However, I feel that it would help to facilitate understanding by moving as much of those details as possible into the main paper so that the reader does not need to refer to the appendix for essential information. For example, it was difficult to understand the experimental design without seeing the stimuli. I would recommend moving the vignettes to the main text so that readers understand what is manipulated.*

We thank the Reviewer for the suggestion and agree that at least vignettes should be shown in the main body of the manuscript. The rationale was to keep the main analysis in the text and put all additional analyses in supplementary materials. Now, we rearranged supporting information and moved the wording of experimental vignettes into the main paper.

*- Were the study and analyses exploratory? If so, this should be stated in the paper.*

Our experimental study is confirmatory since we had particular *a priori* hypotheses, based on which we designed and conducted the experiments. To make it clear for a reader, we directly mentioned that this is confirmatory research in the abstract: “This confirmatory research investigates the influence of risk framing of COVID-19 on support for restrictive government policy based on two web survey experiments in Russia”.

*- Was there a sample size goal? How was the stopping criteria for the study determined?*

As for the sample size goal, we had two different strategies for the first and the second experiments. When we collected the data on a convenience sample for Experiment 1, we used a mailing list of student groups and gave them one week to fill in the questionnaire. Then, we sent them a reminder and waited for another week. The resulting *N* was similar to the sample sizes of other studies conducted on the same convenience sample. For Experiment 2, we used a volunteer online access panel and determined the sample size goal before the fieldwork. Based on the results of Experiment 1 we expected Cohen’s d around 0.15. We set significance criterion at α = 0.05 and statistical power at 80%, and based on anova results we came to the conclusion that minimum 1,100 interviews should be collected and based on t-tests considering that only one factor might be statistically significant (risk severity) we came to the conclusion that minimum 1,400 interviews should be collected. Moreover, 1,500 respondents was the minimum number needed to get a representative study in Russia that would fit our budget. As a result, we collected 1,570 interviews in Experiment 2. We excluded some respondents with low data quality and included in the final analysis 1,438 respondents.

*- In the dependent measures, how were the scale anchors determined? Why was “willingness” measured with a ready/not ready scale? It seems that this wording confuses the acceptance of restrictive measures with the expectation/anticipation of restrictive measures. Please clarify.*

Indeed, there was some mistranslation of the scale and we have corrected it now to 1 – ‘not at all willing’, 5 – ‘fully willing’. We thank the Reviewer for the attention to all details. As for the wording, we asked about the level of support for restrictive measures and we did not ask about their expectations or anticipation, i.e. if they expected some of the restrictive measures or not to be adopted by the government. Some of the restrictive policies had not been adopted by the federal or regional governments but were thoroughly discussed in mass media prior and during Experiment 1. As a result, we asked about the level of support for some of the adopted measures and some measures that were discussed in the media in Experiment 1. For the time when Experiment 2 was conducted, there were some established measures adopted by the government and they were included in the questionnaire. Just to support our claim we give below the exact wording of all our main dependent questions and the scale by which we measured them:

1) ‘How willing are you to sacrifice some of your rights if this helps prevent the spread of coronavirus in Russia?’ measured on a 5-point scale (1 –‘not at all willing’; 5 – ‘fully willing’).

2) ‘To what extent do you support the following measures to prevent the spread of the coronavirus in Russia?’ measured on a 5-point scale (1 – ‘do not support at all’ to 5 – ‘fully support’).

3) ‘To what extent do you support the introduction of criminal liability for violation of the quarantine in Russia?’ measured on a 5-point scale (1 – ‘do not support at all’; 5 – ‘fully support’).

*- Please explain the high 51% drop-out rate with greater detail (e.g., what was the exact percentage of people dropping out on the first page?), were there differences between conditions?*

We have corrected the drop-out rate in the Experiment 1 taking into consideration that 34 interviews were not completed till the end but according to AAPOR standards were included in the analysis as completed interviews. Therefore the droup-out rate was 46%. About half of the dropouts were at the first introduction page (N=309 out of 760). We have clarified this in the manuscript on *p. 10*. We have also checked if there was some difference in breakoff rates among conditions. Overall, 45 dropouts filled in the questionnaire until the treatment randomization. We have found some differences between the treatments in drop out rate: 4 dropouts in the treatment ‘high risk - individual losses’ (drop-out rate = 2%), 21 dropouts in the treatment ‘low risk - individual losses’ (drop-out rate = 10.5%), 8 dropouts - ‘high risk - losses to others’ (drop-out rate = 3.8%), 12 -‘low risk - losses to others’ (drop-out rate = 6.1%). There was no difference between the ‘individual losses’ and ‘losses to others’ treatments in droup-out rates, but some significant differences between the low-risk and high-risk treatments: 8.3% vs. 2.9% respectively, Chi-squared = 11.17, p = 0.001. It is in line with our finding that respondents had a significantly lower level of credibility to the vignettes in the low-risk condition which resulted in a higher drop-out rate in the condition. Though there might be some nonresponse error which results in underestimation of the effect size of the ‘risk severity’ factor, the randomization checks described in the S2 File showed no biases in terms of a number of covariates which make our results of the experiments valid.

*- Statistical details on all tests should be reported. For example, I did not find details related to this statement: “The p-values of joint orthogonality tests indicate that the group differences are insignificant, except for probability of COVID-19 infection in the manipulation of the risk severity factor.”*

Thank you so much for noticing this gap in the report on the statistical analysis. We made sure that statistical details on all tests are reported in the manuscript. As for the example mentioned by the Reviewer, we added references to S2 File, where these p-values are reported.

*- Please proofread the manuscript for typos. For example on page 9: “The vignettes were structured as the set of rubrics with essentially similar content, yet different farming.” P9.*

We thank the reviewer for thoroughly reviewing the manuscript and noticing even minor typos and grammar mistakes. The updated version of the manuscript was revised by a native English speaker in two proofreading sessions.

**Response to the Editor**

*I have now collected three reviews from three experts in the field. All reviewers think that this paper has the potential to make a valuable contribution to the literature, but they suggest several improvements before publication. I am myself familiar with the topic of this manuscript, and I agree with the reviewers. Therefore, I would like to invite you to revise your work for Plos One following their suggestions. On top of their comments, I would like to add one more comment from my own reading.*

*I was surprised not to see a discussion of the literature regarding the effect of proself and prosocial messages on pandemic response, which is very relevant to your research, as you also test the effect of framing a message as a loss to self vs others. There are several works that have explored the effect of proself and prosocial messages; these are the ones I know: Banker & Park (2020), Bilancini et al (2020), Capraro & Barcelo (2020), Heffner et al (2020), Lunn et al (2020), Pfattheicher et al (2020) - but it is possible that there are others (please double check). This line of work is very relevant and I think it should be discussed.*

Thank you very much for your suggestion. In the revised version we discuss previous papers which showed the effect of prosocial messages on pandemic response. We included all papers suggested by the Editor and found some other papers related to this topic. This literature was added on *pp.6-7* in the sub-section “Who is the main object at risk?” and *pp. 30-31* in the Discussion. We have included all papers suggested by the Editor and we have found some other papers.

Thank you for your consideration of the manuscript.

*Sincerely,
Authors*
