# Supplementary material for: The effect of risk framing on support for restrictive government policy regarding the COVID-19 outbreak
Source: PLoS One. 2021 Oct 1;16(10):e0258132. doi: 10.1371/journal.pone.0258132 (PMC8486149; doi:10.1371/journal.pone.0258132)
Supplement: Attachment — Submitted filename: Response_to_Reviewers.docx [file pone.0258132.s011.docx]

*23 July 2021*

Dear Editorial Board,

We again thank all three Reviewers and the Editor for their generous comments and suggestions on the manuscript. We have edited the manuscript to address concerns of the Reviewer #2. In this rebuttal letter we describe the changes made in response to the comments. We also attach the revised manuscript and the revised version of supplementary materials with highlighted changes.

**Response to the Reviewer #2**

*The authors have responded very well and in detail to my suggestions and questions. The revised sections are enlightening and offer interesting additions. Also, I greatly appreciate the newly created supporting material S4, which is helpful to understand measurement and purpose of the considered pre-treatment covariates. A brief note in this regard: I could not find the references cited in S4, hence, it might be helpful to add them directly at the end of the tables in S4. Overall, the paper has been significantly improved and clarified after revising. Therefore, I support and recommend publication.*

We thank the Reviewer for careful reading of the manuscript and the particular attention to the Supporting information. Thanks to this comment, we noticed that there are no references listed for the Supporting information. To address this issue, we updated the Supporting information and added one more file - S9 File – in which the references used in the Supporting information are listed.

**Response to the Editor**

*All reviewers are satisfied with the revision and suggest acceptance. I have selected minor revision in the editorial manager just because one of the reviewers noticed that one reference is missing. Please address this comment at your earliest convenience. I am looking forward to receiving the final version.*

We thank the Editor for the opportunity to revise again the manuscript. We made sure that the References section is complete and added to the Supporting information another file in which the references used in the Supporting information are listed.

Thank you for your consideration of the manuscript.

*Sincerely,
Authors*
